# Possible effects of *Treponema pallidum* infection on human vascular endothelial cells

**DOI:** 10.1002/jcla.24318

**Published:** 2022-03-10

**Authors:** Bibo Xie, Tie Zhao, Sisi Zhao, Jie Zhou, Feijun Zhao

**Affiliations:** ^1^ Institute of Pathogenic Biology and Key Laboratory of Special Pathogen Prevention and Control of Hunan Province Hengyang Medical College University of South China Hengyang P.R. China

**Keywords:** endothelial cells, membrane protein, serum lipoprotein, syphilis, *Treponema pallidum*

## Abstract

Pathogens can affect host cells in various ways, and the same effect can be found in the *Treponema pallidum* acting on the endothelium of host vessels, and the mechanism is often complex and multiple. Based on the existing *T. pallidum* of a cognitive framework, the first concerns involving *T. pallidum* or the bacteria protein directly acted on vascular endothelial cells of the host, the second concerns mainly involved in the process of *T. pallidum* infection in vivo blood lipid change, secretion of cytokines and the interactions between immune cells indirectly. Through both direct and indirect influence, this study explores the role of host by *T. pallidum* infect in the process of the vascular endothelium.

## Graphical abstract

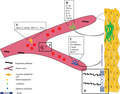

## 1 INTRODUCTION

*Treponema pallidum* belongs to the Spirochaetaceae (spirochetes), ranging from 6 to 15 µm in length, with a diameter of 0.2 µm. It can cause syphilis, a sexually transmitted disease with multiple stages and development that seriously endangers human health, causing 6.3 million new infections each year.^1^ *Treponema pallidum* directly across the mucous membranes of the host or broken skin caused by sexual activity. From a series of previous bioinformatics analyses related to genomics of *T. pallidum*, it can be seen that *T. pallidum* lacks a complete biosynthetic pathway,^2^, ^3^ which may not satisfy its own needs for survival in the host, so *T. pallidum* obtains important or essential macromolecules during the infection of host cells. At the same time, the barrier function of the vascular endothelium of the host is destroyed, allowing it to enter the blood and then spread to the distal tissues and organs through flow of blood.^4^ Therefore, *T. pallidum* attaching to and altering host cells (such as endothelial cells) and extracellular matrix are a key initial steps for infection.

*Treponema pallidum* bind to vascular endothelial receptors (unfortunately, the specific receptors are still unknown) by the bacteria adhesion protein (adhesins such as Tp0751, Tp0136, and Tp0155.) (Table 1) to penetrate tissue barriers, such as the intima of vascular endothelium.^5^, ^6^, ^7^ It has also been determined that TP0326/Tp92 is a *T. pallidum* outer membrane protein, and it is presumed that Tp92 also helps *T. pallidum* attach to host endothelial cells according to the function of homologous protein for mediating cell adhesion.^8^ *Treponema pallidum* then spreads throughout the whole body utilizing blood circulation through tissue and vascular endothelial barriers (e.g., blood–brain barrier and placental barrier), causing multiple organ and tissue infections.^9^ However, it is not clear how *T. pallidum* benefits from invading deep tissue of the gut and musculoskeletal, *T. pallidum* reaches the distant skin and mucous membranes increasing the chances of subsequent transmission. *Treponema pallidum* can affect vascular endothelial function and even cause vascular injury during adhesion and invasion. Clinically, the typical histopathological features of syphilis include vascular injury^10^ and abnormal endothelial cells.^11^ There have been somebreakthroughs in vitro cell culture of *T. pallidum* in recent years.^12^, ^13^, ^14^ Inability to independently culture and genetically manipulate *T. pallidum subsp. pallidum*, the causative agent of this disease, has hindered our understanding of the molecular pathogenesis of pathogens.^15^

**TABLE 1:** *Treponema pallidum* affects endothelial cells

Mycoprotein	Effectors	Effect
Tp0574/Tp47	ICAM‐1	Vascular endothelial cell activation
	RhoA	Increase endothelial permeability
Tp0435/Tp17	VE‐cadherin, F‐actin	Disruption of endothelial cell‐to‐cell Connections.
	ICAM‐1, E‐selectin	Endothelial adhesion
Tp0965	ICAM‐1, E‐selectin, MCP‐1	Increase endothelial permeability
Tp92	TNF‐α, IL‐1β, IL‐6, IL‐8	Damage endothelial cells
Tp1038(TpF1)	IL‐10, TGF‐β, IL‐8	
Tp0751	TNF‐α, IL‐1β, IL‐6	
Tp0155,Tp0483	Bind fibronectin	Adhesion invasion
Tp0751	Fn, ECM proteins	
Tp0136	Bind fibronectin and laminin	

Endothelial cells are highly adaptive because it is constantly aware of changes in the local extracellular environment, such as transient bacteremia, minor trauma, inflammation, or other stresses.^16^ As the immunity in the first line prevents infection, endothelial cells, invaded by bacteria information, give host feedback in a specific manner. Especially, systemic infection of bacteria can change the physiological state of the blood vessels. This change can be presented in several ways, including the death of endothelial cells by directly inducing, weakening for the endothelial cells internal cytoskeleton, undermining the connection between the endothelial cells, or indirectly regulating endothelial function by influencing the immune cells.^17^ This article prefers to focus on exploring the relationship between *T. pallidum* and host endothelial cells, expound and summarize the impact of *T. pallidum* on endothelial function, and explore the relationship between the impact and the relationship between pathogenesis of *T. pallidum*, the diagnosis and treatment of syphilis in the existing cognitive background.

## 2 DIRECT EFFECTS OF *T. PALLIDUM* TO ENDOTHELIAL CELLS

Early research has shown that^18^ vascular tissue cells mainly utilized mucopolysaccharide as a support structure to grow to meet demand. The first step in the interaction between *T. pallidum* and endothelial cells is adhesion, and adhesion protein (such as Tp0751, Tp0136, and Tp0155) can mediate Tp adhesion to human endothelial cells derived from large blood vessels and microvessels.^5^, ^6^, ^7^, ^19^ *Treponema pallidum* in the host for the ongoing metabolism process can use its head end surface of mucopolysaccharide enzyme decomposition glycosaminoglycan, destroy the close connection between the endothelial cells, into perivascular tissue, and further continue to destroy the blood vessels surrounding tissues polysaccharide. The integrity of the support structure makes blood vessel inward or outward, leading to extensive lesions around the blood vessels and blood vessels, thus influencing tissue.^20^ In addition, obstructive endarteritis, periarteritis, and necrosis are further developed, and eventually extensive lesions of blood vessels and perivascular tissues are generated, leading to dysfunction of tissues and organs.^21^

*In vitro* experiments have also found *T. pallidum* to pass through the endothelial cell monolayer without changing the tight connection and found at the intercellular connection.^22^ Some patients with syphilis, such as neurosyphilis, did not find that the blood–brain barrier was damaged, so it was speculated that *T. pallidum* might cross the barrier by regulating the molecular phenotypic changes in host endothelial cells instead of destroying endothelial cells. Multiple proteins of *T. pallidum* are involved in influence for vascular endothelial cells of the body, and these include Human Dermal Microvascular Endothelial Cells (HDMEC), Human Aortic Endothelial Cells (HAEC), Human Umbilical Vein Endothelial Cells (HUVEC), and Human Brain Microvascular Endothelial Cells (HBMEC).^22^, ^23^, ^24^, ^25^, ^26^ Table 1 summarizes the results of multiple studies on the effect of *T. pallidum* expressing proteins on endothelial cells in vitro. Tp0574/Tp47 can not only promote the expression of intercellular cell adhesion molecule‐1 (ICAM‐1) and participate in the activation of vascular endothelial cells,^23^ but also induce the expression of RhoA (a small GTPase protein and its immediate downstream target, Rho kinase [ROCK], control a wide variety of signal transduction pathways) in vascular endothelial cells, activate RhoA/ROCK signal transduction pathway, and induce cytoskeletal recombination, loose intercellular connections, and crevice, resulting in increased vascular endothelial permeability^24^; the present study also provides evidence that TP47 causes an imbalance of matrix metalloproteinase (MMP)/tissue inhibitors of matrix metalloproteinase (TIMP) by increasing the expression and activity of MMP‐1 and MMP‐10 and prompts angiogenesis through Akt/mTOR/S6 signaling in HUVECs.^25^

Tp0435/Tp17 can regulate the expression of ve‐cadherin (cadherin) membrane and F‐actin rearrangement of human vascular endothelial cells, change the connection between endothelial cells, and improve the permeability of vascular endothelial barrier.^26^ Tp0965 can stimulate the expression mRNA and protein of ICAM‐1, E‐selectin, and monocyte chemotactic protein‐1 (MCP‐1) of HUVEC, and decrease the Claudin‐1 expression level of the tight junction protein, leading to increased vascular endothelial cell permeability, which further assists *T. pallidum* in crossing the vascular endothelium.^22^ Recent research results have highlighted that Tp0965 induced Chemerin in endothelial cell dysfunction, which may be involved in the immune pathogenesis of vascular inflammation of syphilis.^27^

## 3 INDIRECT EFFECTS OF *T. PALLIDUM* ON ENDOTHELIAL CELLS

### 3.1 Factor 1: *T. pallidum* infection associated with serum lipoprotein concentrations

The causal relationship between the occurrence and development of syphilis and lipid metabolism is not clear. However, the study^28^ showed that the serum concentrations of high‐density lipoprotein cholesterol (HDL‐C) and apolipoprotein A‐I (ApoA‐I) were decreased in patients with syphilis compared with those in the syphilitic negative control group, while the concentration of apolipoprotein B (ApoB) was significantly increased in patients with syphilis. The main function of HDL‐C is to remove excess cholesterol from the blood and cells. ApoA‐I is one of the main components of HDL‐C, accounting for about 50% of the protein content of HDL‐C. Since ApoA‐I with rare in other lipoproteins, serum ApoA‐I can represent the basic level of HDL‐C. HDL‐C is involved in endothelial cell protection through a variety of pathways,^29^, ^30^ such as endothelial nitric oxide production,^31^ endothelial cell apoptosis,^32^ and endothelial repair after vascular injury.^33^ Like low‐density lipoprotein (LDL), the harmful ApoB of cholesterol‐rich and triglyceride‐rich remains is retained and modified in the arterial wall, leading to vascular damage. Combined with the present study,^34^ the concentration of serum lipoprotein ApoB may be promoting a basic element of vascular endothelial injury. The analysis shows a direct link between ApoB concentrations and low‐density lipoprotein (sd‐LDL) concentrations. The risk of cardiovascular disease is the best predictor of lipoprotein ApoB.^35^ These caused lipid composition, are widely recognized as vascular injury's pathophysiology.^36^, ^37^ For this reason, *T. pallidum* may be thought to affect the vascular endothelium by altering lipid composition, even causing vascular damage.

### 3.2 The second factor: the development of syphilis and the secretion of vascular endothelial cytokines

When the pathogen acts on the vascular endothelium, endothelial cells are activated and releases inflammatory mediators (TNF‐α and IL‐1), chemokines (MCP‐1 and IL‐8), adhesion molecules (ICAM‐1 and vascular cell adhesion molecule‐1, VCAM‐1), clotting factors (PAI‐1), thrombomodulin (TM), etc.^38^ Studies on *T. pallidum* protein stimulating endothelial cells in clinical practice and in vitro^22^, ^23^, ^24^, ^25^, ^26^, ^39^ also agree that *T. pallidum* can induce the secretion of endothelial cells cytokines through a variety of pathways and even affect the vascular structure and function.

In clinical cases, microvascular density and VEGFA expression in dermal lesions of patients with secondary syphilis were significantly higher than normal skin tissues.^39^ Histological sections also showed significant increases in the expressions of Vascular Endothelial Growth Factor A(VEGFA) and ICAM‐1 in the derm‐mucosa of syphilis patients.^40^ The levels of ICAM‐1, VCAM‐1, and IL‐8 were also increased in the rabbit model of syphilis infection.^41^ On the contrary, outer membrane protein Tp92 of *T. pallidum* can induce further elevated levels of the endothelial cell adhesion molecule ICAM‐1 and TNF‐α through Chemerin.^42^ Tp1038/TpF1 has a growth factor‐like activity that induces IL‐8 secretion by activating the CREB/NF‐κB signaling pathway.^43^ Tp0326/Tp92 can promote the secretion of pro‐inflammatory cytokines TNF‐α, IL‐1, and IL‐6 in HMEC‐1 cells and promote the secretion of IL‐8 in HMEC‐1 cells and macrophages through the MyD88/NF‐κB pathway.^44^

**FIGURE 1:**
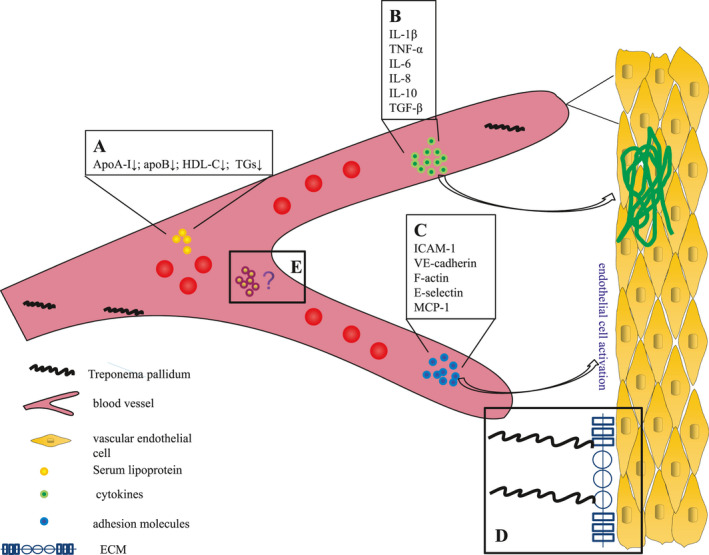
Role of *Treponema pallidum* in endothelial cell phenotype and functions and their direct or indirect targets. *Treponema pallidum* is involved in, (A) serum lipoprotein concentration, (B) cytokines in the inflammatory response to syphilis, (C) vascular endothelial cell activation by cytokines interact with chemokines, (D) binding between pathogens and lipoproteins of the intima, (E) interactions between bacteria, the immune cells, and the endothelial cells

During inflammation, the endothelial cells’ cell phenotype (Figure 1) is activated by major mediators of TNF‐α and IL‐1.^45^ Moreover, endothelial cell activation induces increased vascular permeability and increases the expression of pro‐inflammatory cytokines, chemokines, and adhesion molecules.^46^, ^47^, ^48^ Chemokines, such as MCP‐1 and IL‐8, led to endothelial cells’ inflammatory response by activating the classic NF‐κB pathway.^49^ NF‐κB also regulates cell adhesion molecules, such as ICAM‐1, VCAM‐1, and E‐selectin. Resting expression of adhesion molecules and VEGF, VCAM‐1, and ICAM‐1 is very low, but *T. pallidum* infection leading to endothelial cell activation speculated that involved in the inflammatory factors and endothelial cell adhesion, vessel wall and inflammatory cells infiltration and inflammatory factor accumulation within the immune reaction, the amplification of the inflammatory response can be further activation of endothelial cells, increase the function of endothelial cell damage and damage.^50^, ^51^

### 3.3 Third factor: *T. pallidum*, the interaction between immune cells and endothelial cells

Many studies have shown a close relationship between *T. pallidum* infection and endothelial cells and immune cells, and the response of endothelial cells by *T. pallidum* infection is related to immunity. For example, endothelial cells showed higher adhesion to leukocytes and monocytes by upregulating ICAM‐1, VCAM‐1, and differentiated cluster (CD)11/CD18.^52^ Chemokines released by endothelial cells can recruit large numbers of inflammatory cells to the site of infection, triggering a series of events that lead to an inflammatory response.

Early detection of inactivated spirochetes or non‐pathogenic spirochetes can promote increased adherence of lymphocytes and monocytes to HUVEC.^53^ *Treponema pallidum* reduces leukocyte migration and endothelial permeability across the endothelium, possibly related to immune escape of *T. pallidum*.^54^ Studies have shown that *T. pallidum* regulates the adhesion, permeability, and cytokine secretion of HUVEC in vitro by macrophages exosomes, thereby inhibiting the common inflammatory response of the host and prolonging the survival of the spirochete.^55^ For example, two exosomes containing miR‐216a‐5p and miRNA‐223‐3p, respectively, can suppress the inflammatory response induced by rTp17, miR‐223‐3p is significantly negatively correlated with NLRP3, caspase‐1, and IL‐1β, miRNA‐223‐3p is significantly negatively correlated with IL‐1β, IL‐6, and TNF‐α,^56^, ^57^ and exosomal miR‐146a‐5p is efficiently transported into endothelial cells, reducing monocyte transendothelial migration and endothelial permeability by targeting junctional adhesion molecule C (JAM‐C).^58^ *Treponema pallidum*‐infected macrophages secrete cytokines including ICAM‐1, VCAM‐1, VEGF, and IL‐8. Increased secretion of cytokines and chemokines plays a key role in leukocyte–endothelial interaction, promoting endothelial migration of monocytes, macrophages, dendritic cells, B cells, natural killer cells, and T cells.^59^ These changes may play an important role in the pathogenesis of syphilis. Tp47 can up‐regulate the expression of adhesion molecules ICAM‐1, VCAM‐1, and E‐selectin in HDMEC and promote the adhesion of HDMEC to peripheral blood T cells.^60^ Tp17 also significantly up‐regulated the levels of ICAM‐1 and E‐selectin in vascular endothelial cells, promoted the secretion of MCP‐1 and inflammatory cytokines TNF‐α, enhanced the chemotaxis of HUVEC to monocytes and macrophages, and increased the adhesion rate of THP‐1 cells to HUVEC.^61^ In addition, patients with tertiary syphilis have TpF1‐specific T cells activated by TpF1, which can stimulate HUVEC to secrete IL‐8 and CCL‐20.^62^

Our previous study^63^ showed that Tp92 of *T. pallidum* could induce THP‐1 cells to secrete chemokines of the CXC family, such as IL‐8, CXCL2, and IL‐8/CXCL86, which are also produced by endothelial cells and inflammatory cells and are involved in angiogenesis.^44^ In turn, angiogenesis promotes the metabolism of the proliferative site, allowing more nutrients and energy to flow to the site, which may provide *T. pallidum* with material support for survival, exacerbating the process.

## 4 CLINICAL SIGNIFICANCE AND FUTURE DIRECTION

To sum up, *T. pallidum* infection affects host vascular endothelial cells in various ways, all of which may be closely related, for example, the interaction between lipids and inflammation, the coordination between cytokines and chemokines, and adhesion molecules. These direct and indirect influences may be related to the histopathological characteristics typical of clinical syphilis: vascular injury and endothelial cell abnormalit.^64^ Histological lesions of patients with tertiary syphilis were related to severe arterial wall damage.^65^ The treatment of syphilis is currently dominated by antibiotics.^66^ The patients with third‐stage syphilis have not been adequately treated, and the more common causes of death are aneurysms and thrombosis on atherosclerotic plaques.^67^ However, no studies on *T. pallidum* and/or mycoprotein have confirmed this complex synergistic interaction.

The response of endothelial cells to invasive pathogens is an important predictor of disease severity, and the detection of endothelial function‐related indicators can be used to screen for high‐risk factors of cardiovascular events.^68^, ^69^ Although the effect of spirochete on endothelial cells has only been partially revealed, the significance for *T. pallidum* infection in the pathology of host organs such as blood vessels could be profound. However, changes in vascular endothelial function were not routinely monitored in syphilis patients. Endothelial function testing is used as a marker for early intervention, and it may guide clinical drug therapy and determine whether further symptomatic treatment is needed. Perhaps, we could further explore new therapies for *T. pallidum* infections, for example, by preventing *T. pallidum* from attaching to the endothelium in the first place, or by preventing the endothelium from responding unnaturally to the invading pathogen.

Pathogens can affect the vascular endothelium of the host through a variety of pathways,^69^, ^70^, ^71^, ^72^ with different mechanisms. We note that *Treponema denticola* is a pathogen belonging to the genus *T. pallidum*, which can cause periodontal disease (PD). At present, there is grade A evidence supporting the independent correlation between the occurrence of PD and cardiovascular disease, and the occurrence mechanism is related to the change in serum lipoprotein concentration^72^, ^73^ and the increase in vascular endothelial permeability.^73^, ^74^ Many clinical and basic studies^72^, ^73^, ^74^ have demonstrated that these two elements can affect the vascular endothelium. Therefore, summarizing the available evidence and the study results,^74^, ^75^ it may be reasonable to conclude that *T. pallidum*‐induced vascular injury has a compound cause or possible pathway involving direct and indirect interactions. *Treponema pallidum* lacks a tricarboxylic acid cycle pathway and oxidative phosphorylation component compared with other bacterial pathogens, requiring it to rely on the host for survival.^75^, ^76^ The serum metabolites of syphilis patients showed that the metabolites involved fatty acid biosynthesis, bile acid biosynthesis, ABC transporter, glycerolipid metabolism, choline metabolism, etc. These metabolite changes indicate that *T. pallidum* affects the normal metabolic activity of host cells.^76^, ^77^ In addition, *T. pallidum* may have pathogenic enzymes. For example, Tp34 may play a role in the metal ion homeostasis of the bacteria,^77^, ^78^ and RpoE may play a role in the bacteria's avoidance of the harmful environment of the host.^78^ Nevertheless, whether or not these factors help *T. pallidum* to affect endothelial cell metabolism or damage it needs further testing.

Therefore, the effect of *T. pallidum* on endothelial cells is a complex, multistage, and multipathway process. To further enhance clinical and basic research related to syphilis, it is necessary to elevate the effect of *T. pallidum* on endothelial cells to a causal relationship rather than a simple association. *Treponema pallidum* cannot be cultured in vitro, hindering the research process. In the current study, continuous growth in vitro can be achieved by co‐cultivating Sf1Ep cottontail rabbit epithelial cells in a special tissue culture medium (*T. pallidum* culture medium 2, or TpCM‐2) under microaerobic conditions (1.5% O_2_ and 5% CO_2_).^13^ The parameters affecting the continuous in vitro culture of *T. pallidum* are also listed.^14^ The above may help understand how *T. pallidum* infection causes vascular damage. Causation requires treatment to reduce the risk of complications or be used as a targeted diagnostic indicator of course and efficacy, which requires an effective evaluation of the risk of *T. pallidum* of causing vascular damage to the body during infection. We believe that this is a challenge of great clinical value, and its solution has broad implications for the effective diagnosis and treatment of syphilis.

## CONFLICT OF INTEREST

The authors declare that they have no competing interests.

## AUTHOR CONTRIBUTIONS

Bibo Xie and Tie Zhao made substantial contributions to the conception or design of the work, the acquisition, and analysis; Sisi Zhao and Jie Zhou drafted the work or revised it critically for important intellectual content. All the authors approved the version to be published, and Feijun Zhao agree to be accountable for all aspects of the work in ensuring that questions related to the accuracy or integrity of any part of the work are appropriately investigated and resolved.

## CONSENT FOR PUBLICATION

Written informed consent for publication was obtained from all participants.

## Data Availability

The datasets generated during and/or analyzed during the current study are available from the corresponding author on reasonable request.
